# Prenatal Mouth Movements: Can We Identify Co-Ordinated Fetal Mouth and LIP Actions Necessary for Feeding?

**DOI:** 10.1155/2012/848596

**Published:** 2012-07-02

**Authors:** Nadja Reissland, Claire Mason, Benoist Schaal, Karen Lincoln

**Affiliations:** ^1^Department of Psychology, University of Durham, South Road, Durham DH1 2NR, UK; ^2^Developmental Ethology and Cognitive Psychology Group, Center for Smell, Taste, and Food Science, CNRS (UMR 6265), Université de Bourgogne, 21078 Dijon Cedex, France; ^3^The James Cook University Hospital, Middlesbrough TS4 3BW, UK

## Abstract

Observations of prenatal movement patterns of mouth and lips essential for feeding could have the potential for an assessment of the readiness to feed after birth. Although there is some research on sucking *per se*, we know very little about prenatal preparatory movements for sucking, namely, the ability to co-ordinate opening the mouth widely and then pursing the lips as if around a teat or nipple *in utero*. The purpose of the present study was to test two hypotheses using an adapted version of the Facial Action Coding Scheme: first that mouth stretch (AU 27) will be followed by lip pucker (AU 18), and second that these coordinated movement patterns will increase as a function of gestational age. Fifteen healthy fetuses were scanned four times between 24 and 36 weeks gestation using 4D ultrasound visualization. Results showed a decreased number of mouth stretches with increasing fetal age. Contrary to our expectations, we did not find an increase in movement patterns of mouth stretch followed by lip pucker in preparation for feeding *ex utero*. The results are discussed in terms of sensory triggers *in utero* required to elicit preparatory movements for feeding *ex utero*.

## 1. Introduction

In order to feed from the breast or an artificial teat, new-born infants need to be able to orally grasp and suck. Nutritive sucking has been examined in preterm infants [1] as a means of evaluating developmental risk for poor motor maturity [2]. The difficulty of many preterm infants to suck from the breast or a bottle has been attributed to their underdeveloped motor abilities due to their premature birth [3]. However, others suggest that it might be caused by neurological problems, such as not being able to co-ordinate breathing, sucking, and swallowing [4]. Medoff-Cooper et al. [5] examined the relationship between the pattern of sucking behavior in preterm infants and neurodevelopmental outcomes during the first year of life and found that sucking behavior in preterm infants is an indication of their psychomotor and mental development.

The majority of fetal movement patterns develop during the first half of pregnancy [6] and progress to mature forms after birth [7–9]. Observations of prenatal movement patterns of mouth and lips essential for later feeding ability could have the potential for an assessment of the readiness to suck after birth [10]. Research indicates that by 24 weeks of menstrual age, the fetus responds to palmar stimulation with mouth opening [11]. Humphrey [11] used direct observation of externalized human fetuses placed in a warm fluid bath showing a link between manual and oral activity. In sum, although some research [8–10] has addressed sucking behavior, such as, tongue movements and swallowing, less is known about prenatal preparatory movements for sucking [10], specifically the ability to coordinate mouth and lip movements *in utero*. In one study [10], fetal movements were observed in clusters so that, for example, mouthing was defined as “rhythmic open-and-close mouth movements without significant fluid or tongue movement” (page 68). In contrast to this work, the current study identified two specific movements, namely, the “pursing of lips” (AU 18) which does do not include inferior maxilla movement, whereas “mouth stretch” (AU 27) included a vertical stretching of the mouth by moving the inferior maxilla downward.

A number of studies have documented the development of oro-facial movements in the fetus (e.g., [10–15]), including mouth opening which has been observed in the fetus at 7 to 8 weeks of gestation, sucking at 15 weeks, and swallowing of amniotic fluid at 12 to 14 weeks. Yan et al. ([14]: page 112) defined sucking, observed in 6 out of 10 fetuses once or twice as “a series of movements of the jaws accompanied by the sinking of the cheeks toward the oral cavity, with the fingers always in the mouth.” This contrasts with our observations of the unobstructed fetal face which did not include any stimulation of the oral region. Additionally, some researchers have examined jaw and tongue movements (e.g., [15]). Mizuno and Udea [4] assessed sucking performance in premature infants weekly between 24 to 36 weeks postconceptual age and suggested that the normative data collected on healthy premature infants could serve as a measure to identify preterm infants with sucking difficulties. They reported a significant correlation between premature infants' sucking behavior and performance at 18 months of age on the Bayley Scales of Infant Development which measures infant general psychomotor development. In contrast to investigations on sucking behavior, very little is known about labial and mouth movements essential for sucking and conducive to seizing the breast or artificial teat of a bottle. 

**Figure 1:**
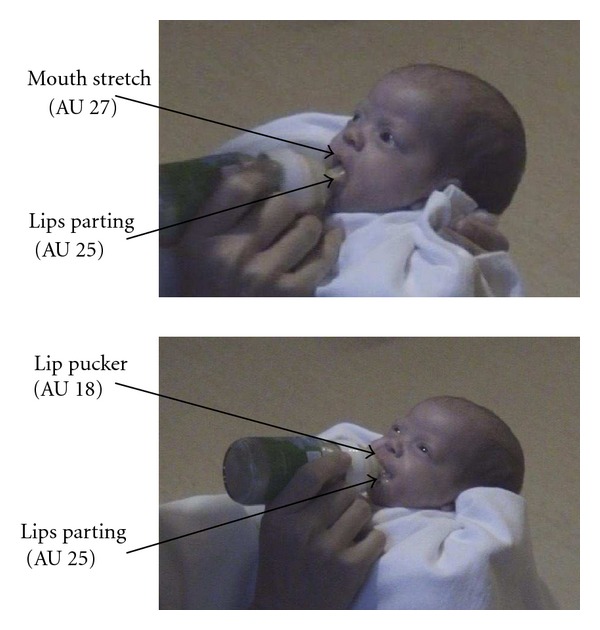
Mouth stretch and lip pucker during feeding in a premature infant aged 35.5 gestational weeks.

**Figure 2:**
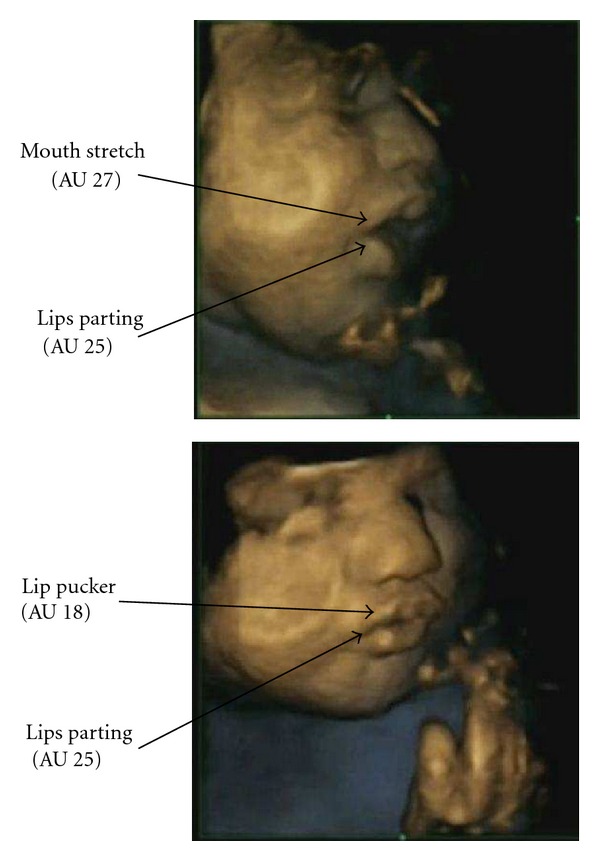
Expression of mouth stretch (AU 27) and lip pucker (AU 18) *in utero* in a fetus aged 33.1 weeks of gestation.

The purpose of the present study was to establish whether we can observe preliminary mouth and lip movements necessary for breast or bottle feeding in fetuses using 4D ultrasound scanning techniques. Movements required to take a breast or bottle include opening the mouth widely and then closing the mouth around the breast or bottle which results in a puckering of the lips (see Figure 1). Hence, if mouth and lip movements are preparatory for mature sucking abilities, we would expect firstly that mouth stretch (AU 27) would be followed by lip pucker (AU 18, see Figure 2) and secondly that these coordinated movement patterns would increase with maturation of the fetus. In order to test this hypothesis, fetuses were observed longitudinally from 24 to 36 weeks gestation in terms of two movements: opening the mouth widely into a mouth stretch and puckering the lips as if closing around a teat. If fetal mouth and lip movements are preparatory for sucking *ex utero*, we would expect as the fetus matures more of these movements to occur and to be coordinated in terms of mouth stretch (AU 27) being followed by lip pucker (AU 18).

## 2. Methods

### 2.1. Participants

Fifteen healthy fetuses, 8 girls and 7 boys, were scanned. The fetuses were observed four times at mean ages of 24.20 (range: 23.9–24.5 weeks), 28 (range: 27.8–28.2 weeks), 32.1 (range: 31.8–32.4 weeks), and 36.1 weeks (range: 36.0–36.4 weeks). All participants were first time mothers with a mean age of 27 years (range: 19–40 years), specifically recruited through the radiographers of the antenatal unit of the James Cook University Hospital, Middlesbrough, UK, following ethical procedures. At birth, the mean weight of the infants was 3283 grams (range: 2380–4160 grams). Mean Apgar scores measured at 1 and 5 minutes after birth were 9.06 (range: 9–10) and 9.33 (range: 9–10). Ethical permission was granted by the County Durham and Tees Valley 2 Research Ethics Committee (REC Ref: 08/H0908/31), James Cook University Hospital. All mothers gave informed written consent.

### 2.2. Procedure for Collecting the Data

After their 20-week anomaly scan had been completed, mothers were approached by a radiographer to seek consent to participate in the study. All participating mothers received four additional scans in the mornings after breakfast, similar in procedure to diagnostic scans they have to undergo at 12 and 20 weeks. During these scans, mothers lay supine either on their back or on the side, depending on the position of the fetus and the comfort of the mother. Fetuses were observed in 4D imaging while active (state 2F or 3F; [16]) for approximately 15 minutes. During consent and before each procedure mothers were made aware that these additional scans were performed for research purposes only and were not routine medical scans. All mothers were given a DVD copy of their scans. 

The fetal face and upper torso were visualized by means of 4D full frontal or facial profile ultrasound recordings, and recorded for off line analysis with a GE Voluson 730 Expert Ultrasound System using a GE RAB4–8L Macro 4D Convex Array Transducer. For each observation period, we coded 10 minutes of scan (which were not necessarily consecutive) when the full face was visible, starting with the first moment when the face was codable.

The Facial Action Coding System (FACS, [17]) developed for adults is an anatomically based system, itemizing facial muscle movements, or ‘‘action units” (AUs). This system was adapted for fetal facial movements [18] using a method designed for the eye brow region of the face [19] which defined movements in the upper face in relation to FACS [17], together with the web resource Artnatomy [20]. Using this new method, we identified two fetal mouth movements required for sucking from breast or bottle, namely mouth stretch (AU 27) “indicating the jaw is pulled down and the mouth is stretched open” ([21]: page 92) and lip pucker (AU 18) “drawing the lips medially, pursing or puckering them, causing the lips to protrude” ([21] page 233). Using Cohen's Kappa, reliability was established for these scans, which were coded independently by a new coder trained in this coding system. This resulted in reliability estimates for AU 27 and AU 18 (mean  =  .87, overall mean range:  .81–.92). 

## 3. Results

All fetuses showed mouth movements during the 10-minute scans. Specifically, for AU 27 (mouth stretch), the fetuses showed a mean frequency in the first scan of 4.40 (range: 0–19), in the second scan of 2.73 (range: 0–9), in the third scan of 3.07 (range: 0–15) and in the fourth scan of  .990 (range: 0–3). A Kruskall-Wallis test (*X* ^2^ = 16.374; df = 3, *P* < .001) showed that in the four scans there was a significant difference in mouth stretch frequencies, with mouth stretch being most frequent at 24 weeks and least frequent at 36 weeks gestation.

In contrast, the frequency of lip pucker (AU 18) did not differ significantly over gestational age. For this AU, the fetuses showed a mean frequency in the first scan of 3.60 (range: 0–20), in the second scan of 3.20 (range: 0–15), in the third scan of 4.87 (range: 0–25), and in the forth scan of 2.47 (range: 0–10). A Kruskall-Wallis test indicated that over the 4 scans there were no significant differences in the frequency of lip pucker displayed (*X* ^2^ = 0.41; df = 3, *P* = .998, ns). 

**Figure 3:**
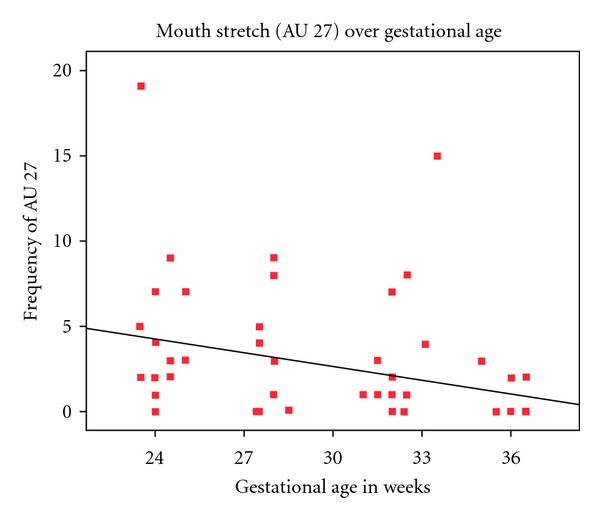
Correlation between the number of mouth stretch (AU 27) and gestational age between weeks 24 and 36. A decrease in mouth stretch frequency can be seen as a function of age.

**Figure 4:**
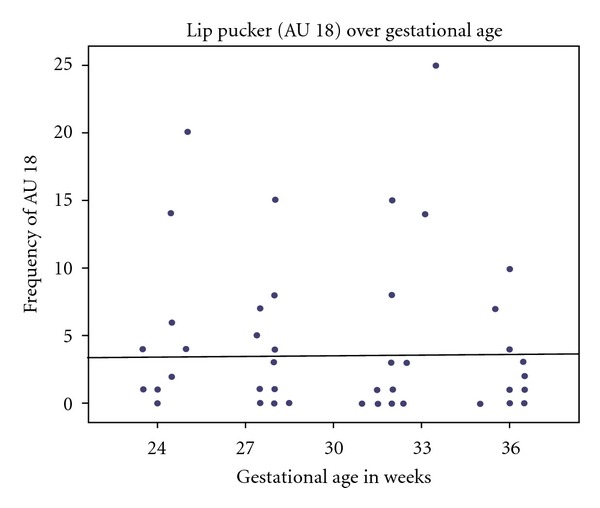
Correlation between the number of lip pucker (AU 18) and gestational age between weeks 24 and 36. Lip puckering frequency is stable as a function of age.

**Figure 5:**
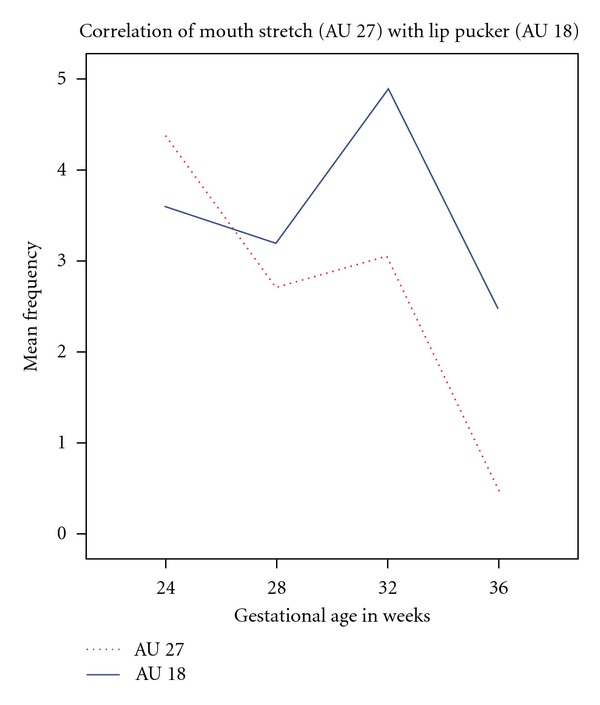
Frequency of mouth stretch (AU 27) and lip pucker (AU 18) observed between 24 and 36 weeks gestation.

**Figure 6:**
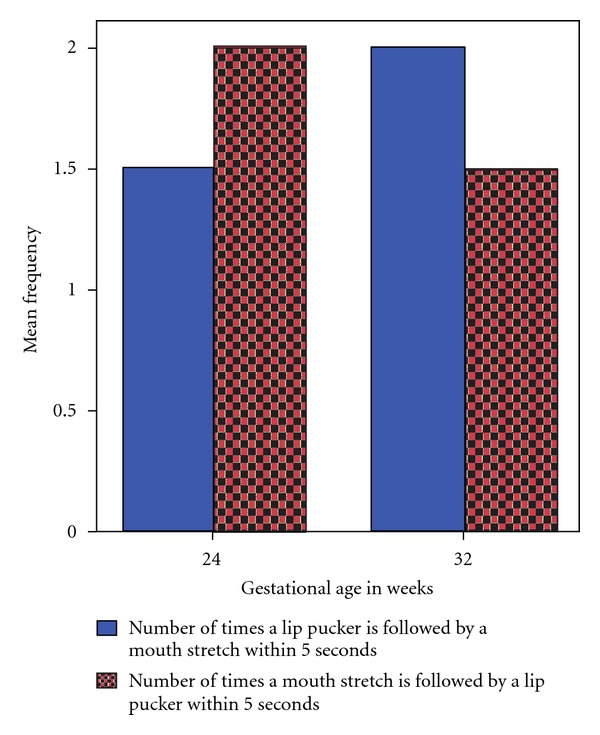
Mean number of mouth stretches (AU 27) followed by a lip pucker (AU 18), or conversely, within 5 seconds of observation in relation to gestational age.

Finally, a correlation analysis indicated that the rate of occurrence of mouth stretch (AU 27) was negatively correlated with gestational age. As the fetuses developed from 24 to 36 weeks of gestation they displayed fewer mouth stretches (Spearman's *ρ* = −.463, *P* < 0.000, Figure 3). In contrast, lip pucker (AU 18) was not significantly associated with age (see Figure 4). Although, mouthstretch (AU 27) and lippucker (AU 18) were correlated (Spearman's *ρ* =  .322, *P* < .005; see Figure 5), fetuses showed very few instances of mouth stretch followed by lip pucker (see Figure 6) and these were unrelated to fetal age.

## 4. Discussion

Research on general fetal movements indicates that with increasing gestation fetuses move less [16]. For example, Kurjak et al. ([22]: page 25), examining facial expressions such as grimacing, mouthing, and yawning, noted “a tendency towards a decreased frequency of observed facial expressions with increasing gestational age.” This is also reflected in the present findings on mouth stretch. Our sample of fetuses observed from 24 to 36 gestational weeks showed decreasing numbers of mouth stretches with increasing fetal age. In contrast, the frequency of lip pucker movements, occurring rather less frequently than mouth stretches, was relatively stable over time. 

Contrary to our hypothesis of an increase in movement patterns of mouth-stretch followed by lip pucker in preparation for feeding *ex utero*, we found only very few instances of the sequence of movements, and these were not related to fetal gestational age. Hence, it seems that the feeding preparatory movement pattern of mouth stretch (AU 27) followed by puckering the lips (AU 18) does not occur more frequently as the fetus grows older. This could be because we observed fetuses moving their mouth without an object stimulating their lips. Given that mothers were observed in the morning after breakfast, although we did not ascertain what they ate before coming to the clinic, the prandial state of the mother is unlikely to have influenced fetal mouth movements differentially. Although Yan et al. [14] observed fetuses while having a finger in the mouth, which could be seen once or twice in 6 out of 10 fetuses, they did not observe a sequence of movements. In order to examine a situation in which the fetus touches his or her lips *in utero* and thereby provides a stimulus akin to a bottle or breast, we currently investigate whether they perform more of the pattern of movements required for oral grasping movements after tactile stimulation. 

Fetal movement patterns which can be seen in the first half of pregnancy [6] develop to mature forms after birth in relation to appropriate stimulation [7]. For example, although certain movements, such as knee jerks, can be observed to occur spontaneously prenatally, they cannot be elicited *in utero* [23]. After birth, however, the infant's vestibular responses to stimulation, such as the Moro response, can be clearly elicited [24]. The sensory trigger mechanism of movement patterns which can be observed to occur spontaneously *in utero* becomes mandatory in the postnatal adaptation of the newborn infant [25]. For example, although the fetus ingests amniotic fluid whenever sucking movements occur [10, 24], after birth sucking behavior needs to be triggered by specific stimuli afforded by the actual feeding situation. Hence, it is a matter of vital biological adaptation that sucking is elicited by touching the lips of the newborn baby in order to initiate feeding [24] and could be the reason why we did not find the expected movement pattern. In contrast, in premature infants, as illustrated in Figure 2, we can observe functional puckering of lips when the teat of the bottle is held in place.

In sum, the present study demonstrates that the movement pattern of mouth stretch (AU 27) followed by lip pucker (AU 18) can be observed prenatally. However, in the absence of appropriate stimulation, we did not observe an increased frequency of these sequential movement patterns with advancing gestation. Future research needs to address the question of whether such a movement pattern can be observed prenatally with stimulation of the oral/nasal region by either touch or chemoreception.
